# FAMoS: A Flexible and dynamic Algorithm for Model Selection to analyse complex systems dynamics

**DOI:** 10.1371/journal.pcbi.1007230

**Published:** 2019-08-16

**Authors:** Michael Gabel, Tobias Hohl, Andrea Imle, Oliver T. Fackler, Frederik Graw

**Affiliations:** 1 Center for Modelling and Simulation in the Biosciences, BioQuant-Center, Heidelberg University, Heidelberg, Germany; 2 Department of Infectious Diseases, Centre for Integrative Infectious Disease Research (CIID), Integrative Virology, University Hospital Heidelberg, Heidelberg, Germany; Imperial College London, UNITED KINGDOM

## Abstract

Most biological systems are difficult to analyse due to a multitude of interacting components and the concomitant lack of information about the essential dynamics. Finding appropriate models that provide a systematic description of such biological systems and that help to identify their relevant factors and processes can be challenging given the sheer number of possibilities. Model selection algorithms that evaluate the performance of a multitude of different models against experimental data provide a useful tool to identify appropriate model structures. However, many algorithms addressing the analysis of complex dynamical systems, as they are often used in biology, compare a preselected number of models or rely on exhaustive searches of the total model space which might be unfeasible dependent on the number of possibilities. Therefore, we developed an algorithm that is able to perform model selection on complex systems and searches large model spaces in a dynamical way. Our algorithm includes local and newly developed non-local search methods that can prevent the algorithm from ending up in local minima of the model space by accounting for structurally similar processes. We tested and validated the algorithm based on simulated data and showed its flexibility for handling different model structures. We also used the algorithm to analyse experimental data on the cell proliferation dynamics of CD4^+^ and CD8^+^ T cells that were cultured under different conditions. Our analyses indicated dynamical changes within the proliferation potential of cells that was reduced within tissue-like 3D *ex vivo* cultures compared to suspension. Due to the flexibility in handling various model structures, the algorithm is applicable to a large variety of different biological problems and represents a useful tool for the data-oriented evaluation of complex model spaces.

## Author summary

Identifying the systematic interactions of multiple components within a complex biological system can be challenging due to the number of potential processes and the concomitant lack of information about the essential dynamics. Selection algorithms that allow an automated evaluation of a large number of different models provide a useful tool in identifying the systematic relationships between experimental data. However, many of the existing model selection algorithms are not able to address complex model structures, such as systems of differential equations, and partly rely on local or exhaustive search methods which are inappropriate for the analysis of various biological systems. Therefore, we developed a flexible model selection algorithm that performs a robust and dynamical search of large model spaces to identify complex systems dynamics and applied it to the analysis of T cell proliferation dynamics within different culture conditions. The algorithm, which is available as an R-package, provides an advanced tool for the analysis of complex systems behaviour and, due to its flexible structure, can be applied to a large variety of biological problems.

## Introduction

Experiments often allow time-resolved measurements of individual components of a biological system. Revealing the most appropriate systematic representation to describe the connectivity and dynamical interactions of these individual components based on such measurements represents a major challenge in order to identify important processes that determine the dynamics. This includes the identification of cell signaling networks based on changing concentrations of molecular components [1, 2], the analysis of key processes for intracellular viral replication cycles [3–5], or the identification of cell differentiation pathways based on the changing concentrations of cells with different phenotypes [6–8]. In most cases, the underlying dynamics and interactions are not completely known and one has to gradually infer an appropriate representation of the system.

**Fig 1:**
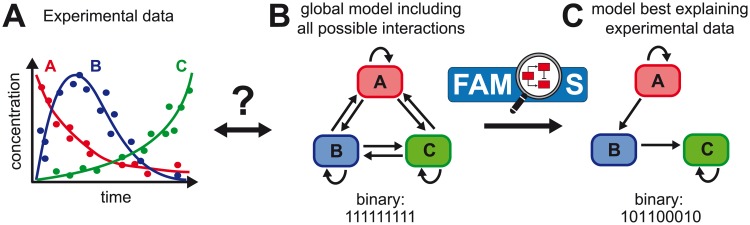
Challenges regarding the identification of the interaction between different components of a biological system. (**A**) Time-resolved measurements for the concentrations of different biological components and factors lead to the question on their dynamical interactions. Out of a global model considering all possible interactions and processes for the different factors within the system (**B**) one needs to identify the (sub-)model that best explains the experimental data (**C**). FAMoS provides a computational method that uses a sophisticated way to search the total model space for the most appropriate (sub-)model.

Mathematical models in combination with information criteria, such as the Akaike information criterion [9], represent a possibility to evaluate the ability of different hypotheses in describing the experimental data. However, complex data structures and large numbers of different components easily result in numerous possible descriptions of their systematic interactions (Fig 1A and 1B). For example, considering a system as in Fig 1B but with 5 different components leads to more than 3.3 × 10^7^ possible models. Despite constant improvement of computational calculation capacities, these bare numbers in possibilities impair their individual testing. Even under the assumption that the evaluation of one model would take only one second, evaluating 3.3 × 10^7^ different models would require more than 1 year in computational run time, and evaluation times will increase with the complexity of the model structure. Most studies address this problem by testing only a reduced number of models, i.e., limiting the number of tested hypotheses based on prior biological knowledge or assumptions [1, 7]. In case such prior knowledge is lacking, only comparisons between different model structures allow a data-driven and unbiased inference of important parameters and processes that determine a systems behaviour. Therefore, there is an increasing need for computational algorithms that efficiently search large model spaces to provide the best systematic description of experimentally observed dynamics.

There have been different implementations of model search and selection algorithms that work reliably well for specific model structures, such as generalised linear models or linear regression [10]. Examples include algorithms presented in the *leaps* [11], *MuMIn* [12], *FWDselect* [13] and *glmulti* [10] packages in R (for a detailed summary and discussion see [10]). In many cases, these algorithms work by a step-wise bottom-up or top-down approach in which parameters are either added or removed from the model to determine their influence on the model performance. However, due to their problem specificity, none of these algorithms can be applied to more complex types of model, such as compartment models based on ordinary or partial differential equations that are widely used in different research areas, as e.g. systems biology or physics. Especially for non-linear type of models, in which many processes interact and might compensate each other, the standard bottom-up or top-down approaches bear the risk of terminating in a local minimum of the model space by predefining the search direction of the algorithm. Although there are a number of model selection algorithms available that are especially designed for the analysis of dynamical systems by providing advanced parameter fitting routines [2, 14], these algorithms partly rely on exhaustive analyses of the whole model space, comparing a predefined number of possible model structures. Therefore, the continuous development of improved model search and selection algorithms [15–19] that are able to handle complex and large model spaces provides an important contribution to various research questions.

Within this line, we developed a Flexible and dynamic Algorithm for Model Selection (FAMoS) that was specifically designed for the analysis of complex systems dynamics within large model spaces, but is also able to handle many diverse mathematical model structures. This flexibility is achieved by letting the user define its own cost function, including a custom fitting routine, for calculating the distance between the model and the data and returning a calculated model selection criterion, e.g. AICc or BIC-values [20–22]. The model selection procedure is based on a dynamical use of backward- and forward search [23] and includes a parameter swap search method that effectively improves the chances of finding suitable models in large model spaces. After validating and testing the robustness and efficiency of FAMoS using simulated data, as well as showing its flexibility to handle different model structures, we applied the algorithm to the analysis of experimental data on the proliferation dynamics of T cells within different environments. Studying CD4^+^ and CD8^+^ T cell dynamics within 2D suspension and 3D tissue-like *ex vivo* cultures, our experimental and mathematical analyses both revealed heterogeneous proliferation dynamics that are influenced by the culture conditions.

## Methods

### General concept of FAMoS

**Fig 2:**
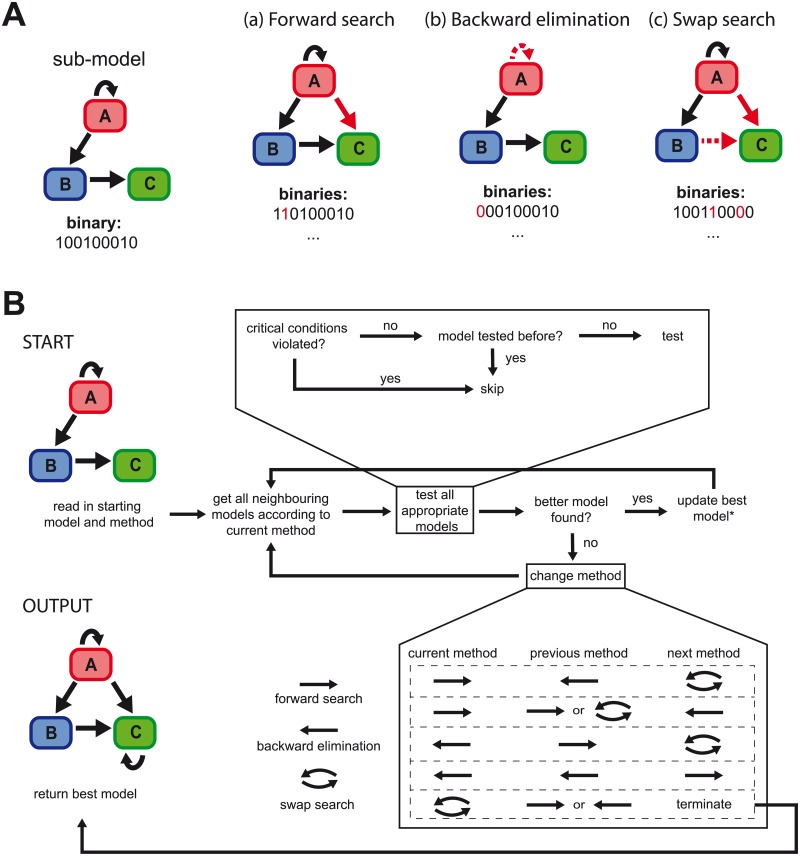
Outline of the algorithm. (**A**) Based on a given (sub-)model of the global model, FAMoS works by creating and testing neighbouring models against experimental data. Thereby, the algorithm distinguishes between three main methods for exploring the model space including (a) *forward search*, in which a parameter that was not considered in the previous model is additionally considered, (b) *backward elimination*, in which a parameter of the current model is deleted from the analysis, and (c) *swap search*, in which two parameters out of pre-defined parameter sets are exchanged. (**B**) Starting with a pre-defined model and method, FAMoS creates the neighbouring models and compares their ability to describe the experimental data, with the methods and/or models dynamically updated at the end of each iteration. The algorithm finally returns the model that was found to best explain the experimental data.

Finding the model that is best able to explain a given set of experimental data out of a multitude of possible interacting components and processes often requires the evaluation of a large number of different models. FAMoS represents an algorithm to efficiently explore the model space and aims at identifying models that incorporate the essential processes and parameters for explaining the observed data. To this end, the algorithm needs a predefined *global* model that contains all possible interactions and processes that are thought to play a role in the observed dynamics. Based on the specific selection algorithm that is outlined in detail below and shown in Fig 2, FAMoS will then evaluate the model space in order to find the (sub-)model that is best able to explain the data. During the selection process, models are fitted to the experimental data by minimizing a cost-function that can be specified by the user. The ability of a specific model in explaining the experimental data is assessed by a standard information criterion, such as AICc, AIC [20, 21] or BIC [22], which is also used for model comparisons (see also [9]).

Due to its flexible structure, FAMoS is applicable to a variety of different model structures. The different elements of the algorithm and their implications are explained in detail in the following. FAMoS is implemented in R [24] and also provided as an R-package via CRAN (https://cran.r-project.org/) along with the necessary documentation.

### Structure of the selection algorithm

Let $\vec{\theta }=\left(\theta_{1},\theta_{2},\ldots ,\theta_{n}\right)$ be the vector comprising all parameters of the global model. Out of this global model, different sub-models can be created by either incorporating (i.e. fitting) or neglecting the processes defined by parameters *θ*_*j*_, *j* ∈ {1, …, *n*}. Each sub-model *m* can be described by a unique binary identifier *α* = (*α*_1_, …, *α*_*n*_), with *α*_*i*_ ∈ {0;1}. Here, *α*_*i*_ = 1 indicates that the corresponding parameter *θ*_*i*_ is estimated, while 0 means the process described by this parameter is either not considered or not allowed to vary, i.e., defined to a default value (see below), within the specific model.

If S describes all possible permutations for *α*, the general aim of the analysis is to efficiently search the model space $\Omega ={\left\{m\left(\alpha \right)\right\}}_{\alpha \in S}$ for finding the model that best explains the observed experimental data.

The main part of FAMoS is the automated, successive generation of new models that are tested against the data. Based on a currently best model, *m*(*α*_best_) (with *α*_best_ ≔ *α*_start_ at the beginning), let *S*_0_ and *S*_1_ denote the corresponding sets of indices for all non-considered and considered parameters, respectively. FAMoS will generate a new set of neighbouring models based on permutations of *α*_best_ according to one of the following methods (Fig 2):

(a) *Forward search*: Add a previously non-fitted parameter to the currently best model, i.e. set *α*_*i*_ = 1, for a particular *i* ∈ *S*_0_. This procedure is repeated to create a new model for every *i* ∈ *S*_0_.
(b) *Backward elimination*: Remove a parameter from the currently best model, i.e. set *α*_*i*_ = 0, for a particular *i* ∈ *S*_1_. This procedure is repeated to create a new model for every *i* ∈ *S*_1_.

In addition to the two methods mentioned above, we implemented a third method which is based on the simultaneous addition of novel and removal of existing parameters of the currently best model, i.e., a replacement of a parameter by another:

(c) *Swap search*: Replace a parameter of the currently best model by another parameter, i.e. set *α*_*i*_ = 1, for an *i* ∈ *S*_0_ and *α*_*j*_ = 0 for a *j* ∈ *S*_1_. Hereby, both parameters have to belong to a predefined swap-parameter set that contains both elements (see below). This procedure is repeated to create a new model for every possible recombination within the predefined swap parameter sets.

All models that are created by one of the three methods are then fitted to the data and their performance in explaining the data compared to the starting model based on a predefined information criterion is assessed. If a better model is found at the end of an iteration, i.e., after evaluation of all newly created neighbouring models, the best model, *m*(*α*_best_), is updated and used for comparison during the next iteration of the algorithm. The current method is kept for the next iteration, with the exception of the *swap search* method which is changed into a *forward search* if the method successfully found a better model (see Fig 2). In case no better model is found based on the current selection method, the algorithm will update its selection method to explore another part of the model space Ω. Therefore, FAMoS keeps track of methods used in the previous iteration and changes them dynamically (see Fig 2). In the end, the best model found over all iterations according to the specified information criterion is returned.

### Definition of critical and swap parameter sets

**Fig 3:**
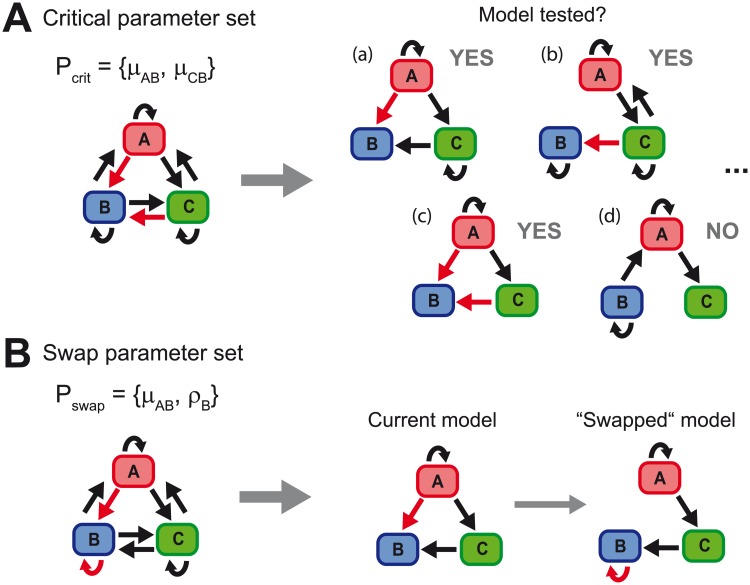
Specific parameter sets. (**A**) Importance of critical parameters sets. In the shown example, model state B is assumed to be achieved initially only from either state A or C. Therefore, the corresponding connections *μ*_AB_ and *μ*_CB_ are defined as a critical parameter set. Only models containing at least one of the parameters are evaluated. (**B**) Definition of swap parameter sets: The transition from A to B, *μ*_AB_, as well as a multiplication within B, *ρ*_B_, are structurally similar parameters that can explain an increase in model state B and define a swap parameter set, i.e., parameters can be replaced against each other for model testing.

FAMoS distinguishes between different parameter sets that can be predefined before analysis. To avoid the testing of models that can already be neglected as they lack important structural parameters, critical parameter sets, $P_{\text{crit}}^{i}\subset {\left\{\theta_{j}\right\}}_{j\in N},\;i\in N$, comprising specific parameter combinations can be defined. Only models containing at least one parameter out of each critical parameter set $P_{\text{crit}}^{i},\;i\in N$ are tested, otherwise these models are discarded (Fig 3A). Critical parameter sets help to account for pre-knowledge on the importance of particular parameters and depend on the model structure. For example, if it is known that one model state can initially only be achieved from one of the other model states (e.g. state B in Fig 3A can initially only be obtained out of state A or C), only models that consider at least one of the necessary connections are worth to be tested.

A second type of parameter sets that can be specified before analysis are the swap parameter sets, $P_{\text{swap}}^{i},\;i\in N$. These parameter sets are essential to define the parameters that can be exchanged by each other when using the *swap search*-method (Fig 3B). If a parameter of a swap parameter set $P_{\text{swap}}^{i}$ is considered within the current model, it can be “swapped”, i.e., replaced, by any other parameter of the particular set $P_{\text{swap}}^{i}$ that is not included in the current model. The swap parameter sets allow to account for structurally similar parameters that can compensate each other due to the model structure. The *swap search* method was developed to swap between these structurally similar parameters in order to effectively avoid termination of the algorithm in local minima of the model space. The algorithm allows the definition of several swap parameter sets. By default, the *swap search* method treats all critical parameter sets as swap parameter sets as according to definition of $P_{\text{crit}}^{i}$ at least one of the parameters of this set has to be present. It is also possible to define the complete parameter set as a swap set, meaning that all parameters can be replaced by each other. However, one has to be aware that in these cases the number of possible neighbouring models can grow quadratically with the number of parameters.

### Cost function and parameter fitting for each model

Each model is fitted to the experimental data by minimizing a predefined cost function. Given a set of experimental observations, the cost function needs to calculate the difference of the model for a given parameter set to the experimental data, e.g. based on a negative log-likelihood function, and needs to return a standard information criterion, i.e. the Akaike (AIC, AICc) or Bayesian information criterion (BIC), to assess model performance (see [9]).

As a default model fitting routine, FAMoS uses the optim-package provided by R. To improve the robustness of parameter estimates, each model can be repeatedly fit with different, randomly sampled starting conditions. During one run, FAMoS will inherit parameter estimates that have been obtained for *m*(*σ*_best_) from previous iterations as one of the starting conditions for subsequent model fits. By this, the algorithm accounts for progressive improvement of parameter convergence during subsequent iterations. To reduce computation time, FAMoS is also able to keep track of its history, i.e., remembering models and their outcomes that have been tested during previous iterations. In this case, models that have been analysed previously are not re-analysed and their previous results are used for comparison. However, implications of this feature are discussed in detail below. Besides definition of critical parameters that have to be included within the individual models, FAMoS also allows the user to specify parameters that will not be fitted and set to default values, as e.g. based on prior knowledge (see options *do.not.fit* and *default.var* in function *famos*). This option allows the user to rely on prior knowledge, as well as implicit model structure conditions, and to reduce the actual model space that will be searched.

### Extensions for parameter fitting methods

As the actual fitting routine represents a crucial aspect for the ability of the algorithm to evaluate individual model performance, the algorithm allows users to specify their own fitting routine within FAMoS. As a default fitting routine, FAMoS uses the optim-function within R with the ability to specify individual fitting methods, such as *Nelder-Mead*, *BFGS* or *SANN*. In our specific example analysing experimental data on cell proliferation dynamics, each (sub-)model tested by FAMoS is fitted to the data using an advanced setup consisting of (multiple) optim-evaluation(s) based on the *Nelder-Mead* algorithm. As this algorithm exhibits—at least in our experience—a very slow convergence speed, we tweaked it in several ways. First, every individual fitting routine is interrupted after 1000 iterations, which in many cases is before optim converges naturally. The resulting parameters are then used as the starting values for the next optim-run, and so on. This process is halted once a specified convergence tolerance has been reached, which in our case defines the difference in the negative log-likelihood between the previous and current optim-run. As the simplexes used in the beginning of an optim-run are larger, halting and re-starting the algorithm effectively covers a broader area in the parameter space. Second, the model fitting routine is repeated several times with randomly sampled initial conditions. In case a better fit is found during one of these runs, the previous fit will be discarded. The user is able to specify the number of runs of optim per evaluation within the *famos*-function (i.e. argument *optim.runs*). While all these tweaks are supposed to help finding a good fit even for more complex models, sometimes a different setup for optim might be more suitable. Therefore, all the options mentioned previously, e.g. the fitting algorithm, step size selection or convergence tolerance, can be redefined by the user.

### Parallelisation

FAMoS is designed to effectively search a large model space by reducing the total number of models that need to be tested. Nevertheless, dependent on the model structure, it still might require the testing of a large number of possibly computationally expensive models in their ability to explain the experimental data. Therefore, FAMoS allows to parallelise the model selection process and to evaluate many different models within one iteration step simultaneously. To this end, the algorithm uses *future*-objects as provided by the *future*-package from Henrik Bengtsson [25] which reduces the total running time of the algorithm drastically if a processing unit with multiple cores or a cluster network is available for computation.

### Selection of a starting model

The choice of the starting model can influence the performance of the selection algorithm as progression through model space is mostly local for each FAMoS run and relies on investigating neighbouring models. Therefore, it is usually recommended to run FAMoS with several starting models to verify the consistency of results. FAMoS provides different options for the selection of the starting model, i.e. using the global model, sampling a random starting model, or starting with a user specified model, e.g., a model containing one parameter out of each critical parameter set. Based on our experience, we generally recommend to verify the consistency by running FAMoS with a certain starting model, and then to perform additional runs by using corresponding complementary models, i.e. by using a model that shows the largest difference to all previously tested models (e.g. by using the built-in option “*most.distant*”). If the individual runs converge towards the same final model, it might serve as an indication of the goodness of the best model.

### Additional materials and methods for the application examples of FAMoS

#### Generation of simulated data for 4 compartment model

**Fig 4:**
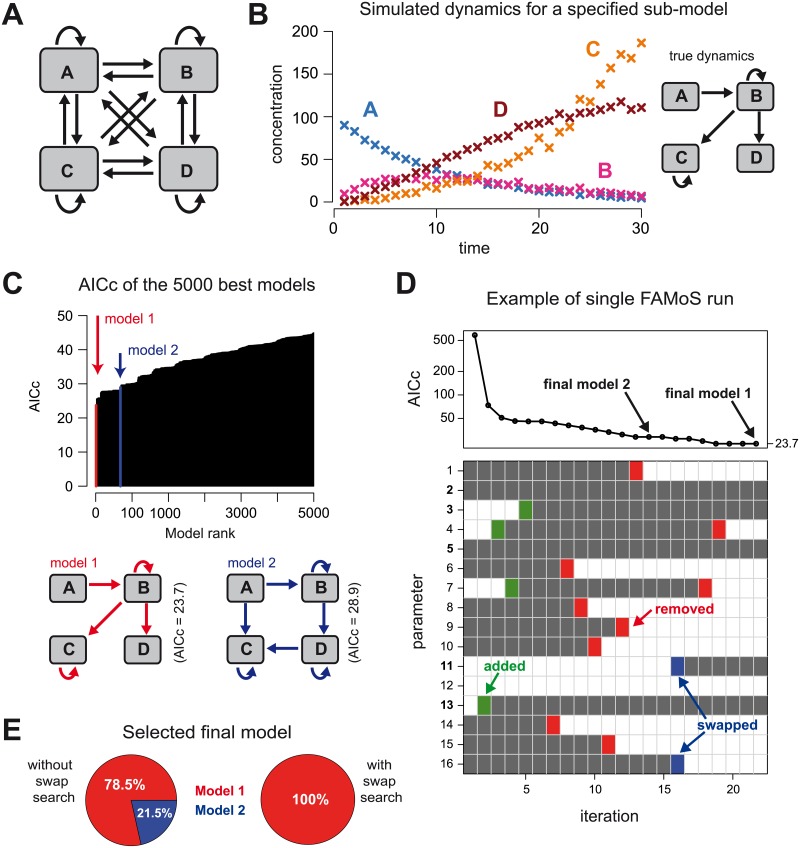
Evaluation of a 4-compartment model using FAMoS. (**A**) The global model containing all possible interactions between and within the 4 different compartments considering multiplication and transition processes. (**B**) Simulated data for the dynamics of the different components using a sub-model as shown on the right (true model). The different crosses indicate the mean over 10 independent measurements at each time point for each compartment (see Methods for model parameterization). (**C**) Evaluation of all possible models of (**A**) against the simulated data with the 5000 best models ranked according to their AICc-values. (**D**) Sketch of a single FAMoS run starting with a specified model and showing the convergence towards the final selected model over time. Individual iterations indicate the changes in the selected models after each iteration by adding (red), removal (green) or swapping (blue) of individual parameters. (**E**) Comparing the performance of FAMoS with and without using the *swap search*-method based on a total of 65.535 runs, i.e., starting FAMoS with every possible (sub-)model of the global model shown in (**A**).

The sub-model describing the interaction between the four different components *A*, *B*, *C* and *D* as shown in Fig 4B is defined by the following system of ordinary differential equations:

$$\frac{dA}{dt}=-\mu_{AB}A$$ (1)

$$\frac{dB}{dt}=\mu_{AB}A+\rho_{B}B-\left(\mu_{BC}+\mu_{BD}\right)B$$ (2)

$$\frac{dC}{dt}=\mu_{BC}B+\rho_{C}C$$ (3)

$$\frac{dD}{dt}=\mu_{BD}B$$ (4)

The parameter *ρ*_*X*_ describes the multiplication rate of the corresponding compartment *X*, while *μ*_*XY*_ defines the transition rate from compartment *X* to compartment *Y*. The system is parameterized with *μ*_*AB*_ = 0.1, *ρ*_*B*_ = 0.1, *μ*_*BC*_ = 0.05, *μ*_*BD*_ = 0.2 and *ρ*_*C*_ = 0.1 using arbitrary units. Initial population sizes are defined by *A*_0_ = 100, while all other compartments are empty, i.e., *B*_0_ = *C*_0_ = *D*_0_ = 0. The system is simulated stochastically using the Gillespie-Algorithm with adaptive-*τ*-leaping [26]. For each of 30 equidistant time points 10 independent simulation runs were evaluated and the compartment sizes were measured to ensure independence between data points.

#### Experimental data

Peripheral blood mononuclear cells served as a source for primary human CD4^+^ and CD8^+^ T cells. Cells were isolated by Ficoll gradient centrifugation from buffy coats of blood donations from healthy individuals and split into three fractions for individual stimulation with 0.5 *μ*g/ml PHA, 5 *μ*g/ml PHA and surface-immobilized anti-CD3 mAb in RPMI 1640 supplemented with 10% FCS, 1% penicillin/streptomycin and 10 U/ml IL-2. After 72 h, fractions were pooled again to result in approx. 95% of CD3 T lymphocytes with typically 70-80% CD4^+^ T cells and 20-30% CD8^+^ T cells. To analyse proliferation, cells were stained with PKH 26 in Diluent C (both Sigma Aldrich), according to manufacturer’s instructions (2.5 × 10^7^ cells in a total volume of 4 ml Diluent C with 2 *μ*M PKH 26 for 2 min at room temperature). Labelling reaction was stopped with FCS for 1 min, followed by washing steps in serum containing medium. Cells were then subjected to suspension and collagen cultures at 1 × 10^5^ cells/100*μ*l as described [27, 28]. Briefly, collagen gels (1.6 mg/ml) were prepared by mixing bovine collagen I (PureCol, Advanced Biomatrix) with bicarbonate-buffered MEM on ice (10*μ*l 10× MEM, 5*μ*l 7.5% NaHCO_3_ and 75*μ*l collagen I). Subsequently, cells were added (45 *μ*l of 3 × 10^6^ cells/ml) to yield a final concentration of 1 × 10^5^ cells/100*μ*l gel and cultures were overlain with 100 *μ*l media, which was exchanged every 2-3 days. Cells were cultured in suspension or collagen for 0, 2, 4 and 7 days. At these time points cells were released by collagenase digestion (100U/100*μ*l culture; 30 min at 37C), washed in PBS and stained to exclude dead cells and distinguish CD4^+^ (CD3^+^, CD8^−^) and CD8^+^ T cells (CD3^+^, CD8^+^) (30 min: dead cell exclusion by fixable viability dye eFluor 450, 1:1000 (ebioscience); 30 min antibody staining: CD8 PE-Vio770, 1:100 (Miltenyi-Biotec), CD3 PE, 1:100 (biolegend)). Cells were fixed with 3% paraformaldehyde in PBS and analysed using a FACSVerse equipped with FACSuite software. Generations were modeled using ModFit LT (Verity Software). For each time point and culture condition, three replicates of the experiment were performed and analysed. Parameter estimates were confined using additional experiments in which the proliferation of CD4^+^ and CD8^+^ T cells was analysed using counting beads (biolegend) for absolute cell counts at the corresponding time points.

#### Parameter fitting for dye-dilution experiment

To fit the individual models describing cell proliferation dynamics to the experimental data using FAMoS, the algorithm relies on a cost-function that calculates the difference of model predictions to the actual data. For each time point *t* the mean, $\bar{y_{i}}\left(t\right)$, and standard deviation, *σ*_*i*_(*t*), is calculated across the individual measurements *y*_*i*_(*t*), with *y*_*i*_(*t*), *i* = 0, 1, …, 9 denoting the frequency of cells that have divided *i*-times until time point *t*. With *m*_*i*_(*t*, *θ*) denoting the corresponding model prediction at time point *t* given parameter set *θ*, the cost-function, $C(\theta ,\tilde{\sigma };y)$, defined as two times the negative log-likelihood neglecting the constant factor log(2*π*), is then given by

$$\begin{array}{c} C\left(\theta ,\tilde{\sigma };y\right)=\sum_{t}\sum_{i}(\frac{{\bar{y}}_{i}\left(t\right)-m_{i}\left(t,\theta \right)}{\sigma_{i}\left(t\right)+\tilde{\sigma }}{)}^{2}+\sum_{t}\sum_{i}\;\text{log}\;\left({\left(\sigma_{i}\left(t\right)+\tilde{\sigma }\right)}^{2}\right) \end{array}$$ (5)

Hereby, $\tilde{\sigma }$ defines an additional factor affecting the empirical standard deviation *σ*_*i*_(*t*) to account for low sample sizes. The parameter $\tilde{\sigma }$ is estimated together with the unknown proliferation and death rates for the particular model, *θ*, and considered as a critical parameter, i.e., each model in the evaluation procedure will contain $\tilde{\sigma }$. Eq (5) is then minimized for *θ* and $\tilde{\sigma }$ using the optim-function with the Nelder-Mead algorithm as described above.

For the analysis, each model during the individual iterations of a FAMoS run is evaluated with 5 different starting conditions. For each cell type, i.e., CD4^+^ and CD8^+^ T Cells, FAMoS is run 5 times with the most distant model compared to the previous model runs used as a novel starting model. The best model according to the corrected Akaike information criterion is returned and shown. Parameter identifiability for the individual parameters of the best model is determined based on profile likelihood analysis [29].

## Results

### Testing the robustness of FAMoS in evaluating the model space

To test the reliability and the robustness of the selection methods implemented in FAMoS, we defined a system comprising four different compartments. We consider multiplication processes within and transmission processes between these different compartments, e.g. resembling proliferation and migration dynamics of cells in different organs or various cell differentiation steps. Considering all possible interactions between the different compartments, with 4 multiplication and 12 transmission rates, we obtain a global model that contains about 6.5 × 10^4^ different sub-models (Fig 4A).

We selected one specific sub-model (Fig 4B) to generate simulated data which were used to assess the qualitative performance of FAMoS in determining the systematic interactions of the different components (see Methods). To this end, we successively used each of the possible sub-models as a starting model for FAMoS and determined the selected model returned by each run of the algorithm. For the first evaluation, we restricted our analysis to the use of the *forward search* and *backward elimination* methods, also neglecting the specification of any critical or swap parameter sets. We found that about 79% of all runs returned the model used for data generation, additionally recapturing the parameter values that were previously used. This model also yielded the lowest Akaike information criterion (AICc = 23.7, model 1) of all tested models (Fig 4C). The remaining 21% of all FAMoS runs all terminated in a different local minimum, which showed a slightly impaired fit of the data (AICc = 28.9, meaning ΔAICc = 5.2 to the best model, i.e., model 1). However, as this second model has two additional parameters compared to model 1 (Fig 4C), the increase in AICc is almost exclusively caused by the more complex model structure as both models capture the simulated dynamics equally well. The vast majority of FAMoS-runs evaluated about 90 different models per run. This corresponds to searching roughly 0.1% of the model space per run which indicates the efficiency of the model selection algorithm.

### Swap search-method increases robustness of the model selection algorithm

To test the ability of the *swap search*-method in improving the analysis of the total model space for finding the most appropriate model, we repeated our analysis of the simulated data with the additional definition of four individual swap parameter sets. If *ρ*_*X*_ denotes the multiplication rate in compartment *X* and *μ*_*XY*_ the transition rate from compartment *X* to compartment *Y*, then the swap parameter set corresponding to cell compartment *A* is defined by $P_{\text{swap}}^{A}=\left\{\rho_{A},\mu_{BA},\mu_{CA},\mu_{DA}\right\}$. With this definition, the set $P_{\text{swap}}^{A}$ combines all parameters that lead to an increase in compartment *A*, which can be explained by multiplication within the compartment, *ρ*_*A*_, and/or influx from one of the other compartments, *μ*_XA_. Thus, these parameters represent structurally similar parameters for compartment *A*. The sets corresponding to the other cell compartments *B*, *C* and *D* were defined accordingly.

Using these swap parameter sets, we re-ran the analysis by FAMoS also including the *swap search*-method. Here, we found that all of the runs which previously terminated in a local minimum (model 2) now ended in the globally best model (model 1), which was also the model used to generate the data, and returned this model as the final result (Fig 4D and 4E). Fig 4D shows that the crucial steps for leaving the local minimum defined by model 2 is the replacement of the parameter *μ*_DC_ by *μ*_BC_ and then the removal of two redundant parameters. Thus, our analysis shows that the *swap search*-method can prevent the algorithm from ending up in a local minimum of the model space and improves the robustness of FAMoS for model selection.

However, the specific choice of the swap parameter sets will influence the performance of the algorithm. For example, using sets defined as $P_{\text{swap}}^{A}=\left\{\rho_{A},\mu_{AB},\mu_{AC},\mu_{AD}\right\}$ (and correspondingly for the other compartments), does not result in the termination in the global minimum for all starting conditions. Therefore, definition of these sets should be done carefully dependent on the structure of the model and potential pre-knowledge of the system. If computationally feasible, the set of swappable parameters can be defined as the set of all available parameter, meaning any two model parameters can be replaced by each other.

The selection of a final model can be further supported by the calculation of the Akaike weights for each parameter across all models evaluated by FAMoS, which provide a quantity for the importance of a parameter for the description of the data [30] (S1 Fig).

### Flexibility of FAMoS for analysing various model structures

The development of FAMoS was especially motivated for the analysis of complex dynamical systems, enabling the exploration of large model spaces. However, due to its flexible structure the algorithm can be applied to a variety of different problems, in contrast to other available model selection algorithms that are specifically designed for one particular model structure [10, 11, 13]. This flexibility is achieved by the ability of the algorithm to allow for individual fitting routines (see Methods), as well as the possibility to incorporate existing toolboxes for model definition and parameter fitting, such as *dMod* [31]. Repeating our analysis of the 4-compartment problem (Fig 4A and 4B) using *dMod* confirmed our results.

As an additional example, we also applied FAMoS to the analysis of generalized linear models (GLM) based on a test problem used in [10, 32]. Re-analzying data to explain the birth weight of babies given as a binary outcome (low, high) based on 8 predictor variables [10], FAMoS found a better model than the one identified by an analysis using the *glmulti*-package in R, which was especially designed for GLM [10] (see *Supporting Information* S1 Appendix). In addition, comparing the run time of both algorithms, FAMoS obtained the result substantially faster with 15 s per run of FAMoS compared to 1-3 minutes per run for *glmulti*.

In summary, FAMoS represents a flexible and user-friendly toolbox to allow for efficient model selection given various model structures.

### Applying FAMoS to the analysis of cell proliferation data

**Fig 5:**
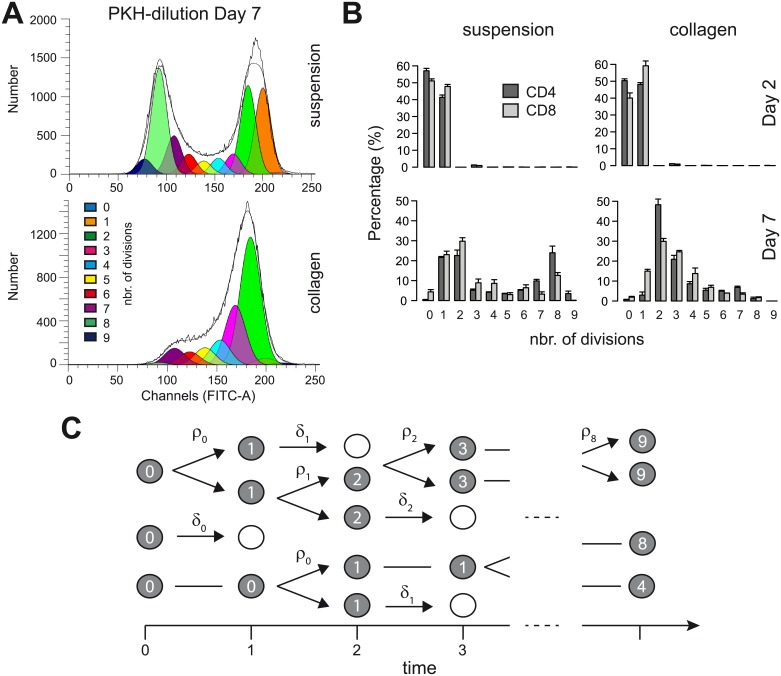
Assessing cell proliferation dynamics by dye dilution experiments within different culture conditions. (**A**) Representative plot for PKH-distribution and separation for cellular subsets according to the number of divisions 7 days after the transfer of the cells. (**B**) Distribution for CD4^+^ and CD8^+^ T cells according to the number of divisions either in 2D suspension (left column) or 3D *ex vivo* collagen cultures (right column) at 2 and 7 days post transfer of the cells. (**C**) Schematic representation of the proliferation dynamics. Cells are assumed to proliferate or die with division dependent proliferation (*ρ*) and death rates (*δ*), respectively.

Having shown the general efficiency, flexibility and robustness of FAMoS, we applied the newly developed model selection algorithm to analyse proliferation dynamics of cells cultured under different conditions. In our experiment, activated CD4^+^ and CD8^+^ T cells were labelled with the fluorescent cellular membrane dye PKH which gets diluted as cells divide (Fig 5A). Transferring 10^5^ cells per 100 *μ*l into either 2D liquid suspension cultures or 3D tissue-like *ex vivo* cultures in which cells are embedded in a collagen matrix [27, 33], cell populations were harvested at different time points and the percentage of cells having undergone a certain number of divisions was assessed by flow cytometry determining their PKH-profile (see Methods for a detailed description of the experiment). Cell proliferation profiles 7 days after the start of the experiment indicated proliferation dynamics which were comparable between CD4^+^ and CD8^+^ T cells, but differed between the different culture conditions (Fig 5B).

Varying mathematical methods have been used to analyse cell proliferation dynamics based on intracellular dye dilution data (reviewed in [34]). These analyses generally indicated division-dependent proliferation and death rates of CD4^+^ and CD8^+^ T cells in cell culture assays [35]. However, as cells can undergo several rounds of division, the identification of the generations at which cell proliferation and/or death rates change, as well as at which point differences between the environments might occur, represents a complex scenario with multiple possibilities. In line with previous analyses [35], we developed a mathematical model that assumed division-dependent cell proliferation, *ρ*_*i*_, and death rates, *δ*_*i*_, with *i* indicating the number of divisions a cell has undergone (Fig 5C). As we are able to distinguish up to 8 individual cell divisions based on the PKH-profile (Fig 5A and [34]), and with *T*_*i*_ indicating the number of cells that have divided *i* times, the corresponding equations are given by

$$\begin{array}{c} \begin{array}{cc} \frac{\text{d}T_{0}}{\text{d}t} & =-\left(\rho_{0}+\delta_{0}\right)T_{0}\\ \frac{\text{d}T_{i}}{\text{d}t} & =2\rho_{i-1}T_{i-1}-\left(\rho_{i}+\delta_{i}\right)T_{i},\;\;\;i=1,\ldots ,9. \end{array} \end{array}$$ (6)

All cells having undergone more than 8 divisions are captured within population *T*_9_. We also included a possible adaptation time *τ*_*E*_ into our model which depends on the environment *E* ∈ {suspension, collagen} and accounts for the fact that cells are transferred into the respective environment for culture. During this first time period after transfer, cells are observed to show a reduced ability to proliferate [27]. Thus, we only allow for proliferation if *t* > *τ*_*E*_ (*ρ*_*i*_ = 0 if *t* ≤ *τ*_*E*_ for *i* = 0, …, 9).

Eq (6) represents the global model with additionally assuming different proliferation and death rates for cells in suspension and 3D collagen environments. With 42 different parameters (10 proliferation, 10 death rates and one adaption time for each environment), we end up with a total set of more than 10^12^ different possible models for each cell type, i.e., CD4^+^ and CD8^+^ T cells. Even if we assume that the evaluation of one model would only take 1 second in computational run time, evaluating all possible models would require more than 31,000 years on a single computer. Therefore, we used FAMoS to fit Eq (6) simultaneously to the experimental data of the different environmental conditions in order to determine the most appropriate model structure describing the observed cellular turnover, and to detect possible differences in the dynamics between the different environments.

For the analysis, each generation-dependent proliferation rate, *ρ*_*i*_ inherits its value from the previous generation if the algorithm does not select for a model with a change in one of these parameters for a particular generation, i.e, *ρ*_*i*_ = *ρ*_*i*−1_, *i* ∈ {1, …, 9} if the parameter *ρ*_*i*_ is not selected to be fitted within the model. Similarly, parameters in suspension (S) are considered to be the same as for collagen (C) until the algorithm selects for an environmental difference in a particular generation, i.e., $\rho_{i}^{S}=\rho_{i}^{C}$, ∀*i* < *j* if the model selects for a difference between the environments to appear after generation *j* ($\rho_{j}^{S}\neq \rho_{j}^{C}$). The same holds true for the generation-dependent death rates, *δ*_*i*_. We define $P_{\text{crit}}=\left\{\rho_{0}^{C},\delta_{0}^{C}\right\}$ as a critical parameter set as the initial cell population starts in generation 0. Furthermore, the algorithm is allowed to swap between any parameters. In summary, the analysis of the cell proliferation dynamics within different environments by FAMoS utilizes the definition of critical and swap parameter sets in order to improve model selection analysis.

### Cell culture conditions influence cell proliferation dynamics

**Fig 6:**
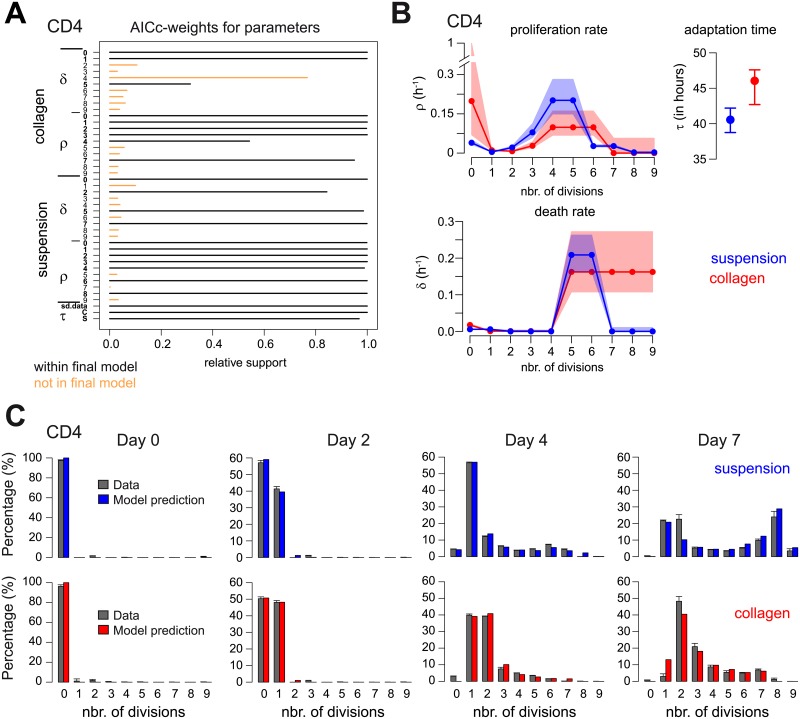
Assessing cell proliferation dynamics of CD4^+^ T cells by dye-dilution experiments within different culture conditions. (**A**) Akaike weights for the individual parameters based on all 8669 model evaluations within the 5 independent FAMoS runs performed for the analysis of the CD4^+^ T cells. Parameters selected within the final model (depicted by 2 out of 5 runs) are shown in black, non-selected parameters in orange. A representative FAMoS run is shown in S2 Fig. (**B**) Determined model structures and parameter estimates for the generation-dependent proliferation (*ρ*) and death rates (*δ*), as well as adaptation time periods (*τ*) for CD4^+^ T cells within suspension (blue) and collagen (red). Points indicate best parameter estimates obtained for each rate with shaded areas defining the corresponding 95%-confidence intervals obtained by profile-likelihood analyses (see Table 1 and S3 Fig). (**C**) Observed (grey) and predicted distribution of cell populations for CD4^+^ T cells using the determined model structure and the best parameter estimates obtained for each condition (blue = suspension, red = collagen).

For each cell type, we used 5 subsequent FAMoS runs always taking the most distant model to all previously tested models as the starting condition for a new run (see Methods for a detailed explanation of the fitting procedure). Each FAMoS run comprised on average 35 (range 19-50) iterations with ∼1700 (range 559–2516) models tested per run (see S2 Fig for an example of a single FAMoS run). For CD4^+^ T cells, 2 out of 5 FAMoS runs (run 4 and 5) converged towards the same final model, with the results of the other 3 runs only showing minor differences in the determined parameter structure. This particular model had the lowest AICc-value across all 8669 models tested within the 5 independent FAMoS-runs with the selected parameter structure also getting the largest support based on the combined Akaike weights (Fig 6A). With a total of 8669 (8490) models tested within the 5 independent FAMoS runs for CD4^+^ (CD8^+^) T cells, the analysis captured roughly 10^−6^% of the global model space.

The determined model structure indicates dynamic changes within the generation-dependent proliferation and death rates for CD4^+^ T cells (Fig 6). Within suspension cultures, the changes in proliferation rates can be roughly distinguished into 4 different phases: Following a first decrease after the first division (*ρ*_0_ = 0.039(0.032, 0.05)h^−1^ vs. *ρ*_1_ = 0.004(0.003, 0.005)h^−1^) proliferation rates gradually increase until reaching a peak after around 4-5 divisions (*ρ*_4-5_ = 0.20(0.14, 0.28)h^−1^). The proliferation rate then substantially decreases within the following generations to roughly 10% of the value observed at the peak (*ρ*_6−7_ = 0.027(0.023, 0.032)h^−1^) before stabilizing at a low level (*ρ*_8−9_ = 0.003(0.001, 0.005)h^−1^) (Fig 6B and Table 1, numbers in brackets represent 95%-confidence intervals of estimates obtained by profile likelihood analysis of the final model). The corresponding death rates indicate similarly 3-4 different phases with the largest death rates during the generations where the proliferation rates peak simultaneously (Table 1).

**Table 1:** Estimated generation dependent proliferation and death rates. The table shows the best estimates for the generation dependent proliferation, *ρ*, and death rates, *δ* (both in ×10^−2^h^−1^), as well as the length of the adaptation phase (in h) for both CD4^+^ and CD8^+^ T cells based on the cell proliferation data and dependent on the culture condition. Numbers in brackets denote 95%-confidence intervals obtained by profile likelihood analysis (upper bound of 1 indicates unidentifiability). Estimates are obtained for the best model identified by FAMoS for each cell type indicating influences of the environment on cell proliferation. In addition, standard deviation of the data, $\tilde{\sigma }$ (in ×10^−3^), was estimated with ${\tilde{\sigma }}_{\text{CD}4}=8.7\;\left(7.1,11.1\right)$ and ${\tilde{\sigma }}_{\text{CD}8}=2.5\;\left(1.8,3.8\right)$. A visualization of the estimates can be found in Fig 6B and Panel B in S5 Fig.

CD4^+^ T cells				CD8^+^ T cells			
Suspension		Collagen		Suspension		Collagen	
generation	estimate	generation	estimate	generation	estimate	generation	estimate
*ρ*_0_	3.94 (3.26, 5.00)	*ρ*_0_	19.8 (7.0, 100)	*ρ*_0_	100 (68.2, 100)	*ρ*_0_	100 (68.8, 100)
*ρ*_1_	0.42 (0.37, 0.47)	*ρ*_1_	1.07 (0.99, 1.18)	*ρ*_1_	0.61 (0.54, 0.68)	*ρ*_1−2_	1.47 (1.30, 1.68)
*ρ*_2_	2.13 (1.65, 2.70)	*ρ*_2_	0.71 (0.60, 0.88)	*ρ*_2_	0.97 (0.79, 1.17)		
*ρ*_3_	7.91 (5.85, 11.25)	*ρ*_3_	2.78 (1.96, 4.25)	*ρ*_3_	4.99 (3.80, 6.60)	*ρ*_3−4_	1.15 (1.02, 1.32)
*ρ*_4−5_	20.2 (14.9, 28.2)	*ρ*_4−6_	9.84 (6.8, 16.1)	*ρ*_4_	2.21 (1.37, 3.49)	*ρ*_5−7_	2.02 (1.49, 2.58)
				*ρ*_5_	9.40 (5.20, 22.1)		
				*ρ*_6_	1.23 (0.79, 2.00)		
*ρ*_6−7_	2.68 (2.30, 3.20)	*ρ*_7−9_	0.0 (0.0, 5.70)	*ρ*_7_	5.29 (3.10, 12.70)		
*ρ*_8−9_	0.28 (0.10, 0.49)			*ρ*_8−9_	0.02 (0.0, 0.12)	*ρ*_8−9_	0.04 (0.0, 0.96)
*δ*_0−1_	0.58 (0.53, 0.62)	*δ*_0_	1.74 (1.60, 2.05)	*δ*_0_	0.40 (0.25, 0.56)	*δ*_0_	0.79 (0.51, 1.11)
*δ*_2−4_	0.0 (0.0, 0.35)	*δ*_1−4_	0.0 (0.0, 0.30)	*δ*_1−3_	0.0 (0.0, 0.04)	*δ*_1_	0.0 (0.0, 0.25)
*δ*_5−6_	20.9 (16.2, 26.3)	*δ*_5−9_	16.2 (10.7, 27.3)	*δ*_4−6_	5.36 (3.60, 7.60)	*δ*_2_	0.81 (0.37, 1.29)
*δ*_7−9_	0.0 (0.0, 1.08)			*δ*_7−9_	0.0 (0.0, 2.00)	*δ*_3−9_	3.14 (2.36, 4.03)
*τ* (in h)	40.6 (38.7, 42.2)	*τ* (in h)	46.1 (42.7, 47.6)	*τ* (in h)	47.6 (46.4, 47.6)	*τ* (in h)	47.5 (47.2, 47.5)

We find that the observed differences in the proliferation dynamics between the different culture conditions (Fig 5B) can largely be explained by a slightly reduced proliferation potential of cells in collagen while the dynamics of the rates follows a similar 4-phase pattern as observed in suspension (Fig 6A). In addition, our algorithm particularly selects for models with differences in the length of the adaptation phase between the two culture systems. Thereby, the length of the adaptation phase is estimated to be slightly shorter in suspension compared to collagen (*τ*_*S*_ = 40.6(38.7, 42.2) hours vs. *τ*_*C*_ = 46.1(42.7, 47.6) hours, numbers in brackets represent 95%-confidence intervals of estimates). This observation agrees with the experimental conditions as cell originate from suspension cultures before being transferred into their respective culture conditions. Despite the complexity of the selected models, nearly all individual parameters, except for *ρ*_0_ in collagen, are identifiable (see Table 1 and S3 Fig). Model predictions based on the best estimates (Table 1) agree with the observed distributions for CD4^+^ T cells for both environments (Fig 6C), with obtained parameter estimates supported by the Akaike weighted parameter estimates of all evaluated models (S4 Fig).

For CD8^+^ T cells, we also find dynamic changes within generation-dependent proliferation and death rates (S5 Fig, Table 1). However, a similar 3-4 phase dynamics as observed for CD4^+^ T cells is less pronounced for this cell type, which is mainly due to the fact that the proliferation rate of the first generation, *ρ*_0_, is not identifiable for both environments (S6 Fig). This leads to less reliable parameter estimates for this cell type (S6 and S7 Figs).

However, given the comparable proliferation structures identified for CD4^+^ and CD8^+^ T cells, our analysis indicates an influence of the culture condition on the cell proliferation dynamics with cells in 3D collagen showing impaired proliferation capacities.

## Discussion

Finding the system that explains observed data out of a multitude of possible scenarios remains a challenging and computationally intensive task. Therefore, the number of candidate models in studies is usually limited to allow for timely evaluations and comparisons, as for example for the identification of cell signaling [1] or cell differentiation pathways [7]. However, pre-selection of models can bias results and miss appropriate model structures as only a set of models is evaluated against the data. With FAMoS we present a simple and flexible method to efficiently evaluate a global model space without the requirement for model pre-selection. FAMoS was especially designed for the analysis of complex systems, including models based on differential equations, which are characterized by large model spaces. The presented R-package containing FAMoS provides a framework that allows for easy parallelisation of analyses on high-performance computing platforms and includes several options that aim at improving the model selection routine if prior-knowledge on the underlying model structure is available.

FAMoS extends previous data-oriented model selection tools that either perform exhaustive analyses of the total model space or use one-directional search methods, e.g. by increasing or decreasing model complexity by adding or removing parameters, respectively. While analysis of all models could be hindered simply due to the large number of possibilities, the latter procedure might bias results, especially if models contain structurally similar parameters. To reduce the risk for the search algorithm to end up in a local minimum of the model space, we implemented a dynamic forward-backward search and added a new method for model selection within FAMoS, called the *swap search*-method. While this method is able to significantly improve the fitting results by increasing the robustness of selecting an appropriate model (see Fig 4E), a meaningful application of this feature requires some knowledge of the underlying model structure analysed (see Results). The *swap search*-method was originally inspired by the problem of finding cellular differentiation pathways using cell population measurements [6]. As these kind of data combine cell proliferation, differentiation and migration dynamics, model systems describing the dynamics have to account for many structurally similar parameters that impair parameter identification. These structurally similar parameters can be grouped into different sets, which allows FAMoS to find the most reasonable model system despite parameter similarity (see example for Fig 4E). In general, if the total number of model parameters is not too large, or if sufficient computational power is available, it is also possible to define the swap set as the set containing all model parameters. This would allow an unbiased use of the *swap search*-method, especially if no prior-knowledge on structural similarities between model parameters is known. However, such a choice would increase computational run time which might be unfavorable in case of large model systems.

As it is the case for almost all minimization routines, FAMoS cannot guarantee that the result returned from one particular run is truly the globally best model. Since the basic model selection methods (*forward search* and *backward elimination*) are local search methods, the chances of ending up in a local minimum of the model space might be very high. Although the newly developed *swap search*-method reduces this risk (see Fig 4D and 4E), it will not eliminate the possibility of ending up in a local minimum completely. Therefore, re-running FAMoS with different starting models is a reasonable approach to improve the reliability of the performed analyses. In order to generate a comprehensive search of the total model space, the FAMoS-package provides an option to use a starting model that is least similar in terms of used parameters to all models tested previously (*get.most.distant*). This allows to start the new selection process in a different area of the global model space and, thus, provides an extended search of the total model space.

We followed this approach when analysing the cell proliferation dynamics of CD4^+^ and CD8^+^ T cells based on dye-dilution data by using 5 subsequent FAMoS runs. Assuming a model that accounts for generation-dependent proliferation and death rates, we identified a roughly three- to four-phase proliferation dynamics for T cells in 2D suspension cultures, which was particularly visible for CD4^+^ T cells (Fig 6B, S8 Fig). Although being in line with previous observations [35], the number of divisions after which these changes could be identified differ from the generation marks determined by Yates et al. [35]. This is potentially influenced by the fact that our analyses allowed for more degrees of freedom, i.e., generation-dependent proliferation and death rates are allowed to change independently, in comparison to the analysis by Yates et al. which assumed both rates to change simultaneously. Cells in 3D *ex vivo* collagen cultures were found to follow a similar phase-dependent proliferation dynamics (Fig 6B, S8 Fig). However, both, the experimental data itself (Fig 5B), as well as the mathematical analyses indicated a slightly reduced proliferation capacity of cells in collagen compared to suspension. Thus, extracellular factors, such as local cytokine concentrations and profiles, that are shaped by the individual culture conditions impact the cell proliferation dynamics. While in suspension cultures cells sediment due to gravity at high density at the bottom of the culture dish, the 3D collagen matrix provides a scaffold that results in 3D spacing of the cells. This increased dispersion of the cells would consequently result in diminished local cytokine concentrations and reduced cell interactions that might impair individual cell proliferation potential [36]. This difference within the proliferation capacity for the two culture conditions is also reflected in the estimation of a slightly longer adaptation phase for cells in collagen compared to suspension (46.1 vs. 40.6 hours, Table 1). The consideration of such an adaptation phase, in which transferred cells show an impaired ability to proliferate while adapting to the novel environment, is strongly recommended by the evaluations of the algorithm (Fig 6A). This finding is irrespective of the culture condition and was already noticed before [35]. Our estimates for the length of the adaptation period are slightly larger than the values found by Yates et al. [35] (∼40-47 hours vs. 36 hours), which also leads to a higher proliferation probability for cells for the first division (*γ*_0_ ∼ 0.32 − 0.45, calculated numbers represent the probability for a cell to divide within a 12h-time step comparable to [35], i.e., $\gamma_{i}=1-e^{-12\rho_{i}}$ with taking the boundaries of the 95%-CI of the estimates into account (Table 1)). Despite differences in the actual numbers, both analyses (here and [35]) find peak cell proliferation probabilities for cells after they have undergone an intermediate number of divisions (*γ*_4−5_ ∼ 0.83 − 0.97 vs. *γ*_1−3_ = 0.41 − 0.43 in [35]).

Besides arguing for generation-dependent proliferation dynamics, our mathematical analysis could also support the existence of cell populations that are heterogeneous in their proliferation potential [37]. Cell division rates have been found to be largely correlated within individual lineages [38–40]. In the modeling framework used here, this heterogeneous proliferation potential of individual lineages will be reflected within the dynamic changes of the generation-dependent proliferation rates. With the cell proliferation probabilities being found to be highest at intermediate generations, our analysis arguably supports the existence of two distinct cell populations with high and low proliferation potential [37]. Given the similar pattern of the identified proliferation rates for cells in suspension and collagen (S8 Fig), our results could indicate that culture conditions might largely influence the total proliferation potential rather than an increase in the heterogeneity of the proliferation potential. Additionally measuring individual T cell clone sizes, as well as accounting for cell populations with different proliferation potentials [35], or for the dependence of division and death rates on the time since the last division of a cell within the model structure [41–43], might improve the quantification and characterization of the observed proliferation dynamics.

As a general aspect of model selection algorithms, it needs to be mentioned that the “best” model returned by FAMoS does not necessarily represent the “true” model that generated the observed data. FAMoS, as well as most other model selection algorithms, work by comparing models based on standard information criteria, such as the AIC, AICc or BIC [9, 20–22]. These information criteria have been interpreted as determining the model with the least deviation from the true system (AIC, AICc) or with the highest likelihood comparing several models (BIC) [30]. Each of the information criteria has their advantages and disadvantages, and the particular differences and individual assumptions have been discussed elsewhere [9, 44, 45]. The used information criteria rely on the likelihood of the model with regard to the data thereby penalizing for the complexity of the model. As such the ability of the information criterion to detect the “best”/“true” model relies on the assumption that the algorithm returns the best fit for each of the tested models and, therefore, depends on the performance of the fitting routine. However, especially for more complex models this assumption might not hold true. FAMoS features several options to increase the likelihood of finding a reasonable fit, including refitting of previously analysed models, parameter inheritance for subsequent iterations and the consideration of different fitting methods (see also Methods). Several existing model selection algorithms already provide advanced fitting methods for the challenging analyses of dynamical systems while relying on model comparisons across the whole model space [2, 14, 17, 18, 46]. Extending FAMoS by combining the developed search strategies with these elaborated fitting routines might additionally improve robustness of the estimation and, thus, selection procedures. In addition, instead of just relying on the single final model returned by FAMoS, it is recommended to determine the importance of individual parameters for explaining the given data, e.g. based on the Akaike weights calculated across all tested models [30], which allows a more reliable identification of appropriate model structures (S1 Fig, Fig 6A).

There are already a number of pre-existing algorithms available that perform model search and selection based on different methods, most of them suited to address a specific model structure [10–12]. As such, the currently available packages are designed to handle the model selection process for their given model structure as efficiently as possible. Additionally, no evaluation function needs to be specified in these packages, making them more accessible for less experienced users. The here presented FAMoS-package is not intended to replace these previously established R-packages. FAMoS with its flexible design rather fills a gap to allow selection on more complex model structures and large model spaces that is not efficiently possible based on the existing algorithms. Additional algorithms have been developed recently that work on the identification of dynamical systems using sparse regression [15], also in combination with information criteria [16]. Kaltenbacher et al. [19] presented an algorithm based on forward search and backward elimination that showed convergence for finding sparse solutions of inverse problems, but without the here newly developed swap-search method that allows to account for prior-knowledge of the analysed system and improves evaluation of the total model space. The algorithm published by Mangan et al. [16] works remarkably well in recovering the true underlying dynamics for different theoretical examples using dynamical systems of varying complexity. However, it remains to be noted that the successful model identification in these examples relied on a frequent and dense sampling of the system components, and also neglected systems with possible compensating effects of parameters (as e.g. cell migration and proliferation dynamics in Fig 4A). Therefore, the ability of FAMoS, as well as other model selection algorithms, to determine the “true” dynamics also depends on the quality of the sampled data used for analysis. Dependent on the data, i.e, frequency and time points of sampling, as well as signal-to-noise ratio, several models could potentially explain the observed dynamics, especially if these models are structurally similar. FAMoS is designed to account for prior-knowledge within the search and selection process by the definition of critical and swap parameter sets, which can help to disentangle structural similarities.

In summary, with FAMoS we present a flexible, customized toolbox for model selection that is applicable to a large number of different scenarios. It is especially useful and designed for the analysis of complex systems, allowing the efficient evaluation of a high-dimensional model space. The application to cell proliferation data in this study, as well as the successful application of an earlier version of the algorithm to the analysis of CD8^+^ T cell differentiation and migration dynamics between different organs [6], shows its potential for addressing various biological problems. As such, FAMoS provides a useful method for data-driven analyses in systems dynamics.

## Supporting information

S1 Fig Akaike weights for the individual parameters within the 4-compartment model. (PDF)

S2 Fig Exemplary FAMoS-run analysing CD4^+^ T cell dynamics. (PDF)

S3 Fig Profile likelihood analysis for parameter estimates of CD4^+^ T cells. (PDF)

S4 Fig Distribution of parameter estimates for CD4^+^ T cells considering different model structures. (PDF)

S5 Fig Model selection and parameter estimates for CD8^+^ T cell dynamics. (PDF)

S6 Fig Profile likelihood analysis for parameter estimates of CD8^+^ T cells. (PDF)

S7 Fig Distribution of parameter estimates for CD8^+^ T cells considering different model structures. (PDF)

S8 Fig Net-proliferation rates for different generations. (PDF)

S1 Appendix FAMoS analysis of generalized linear models. (PDF)

S1 Table Net-proliferation rates for different generations. (PDF)

S1 Dataset Experimental data of CD4^+^ and CD8^+^ T cell proliferation. (XLS)
