# Supplementary material for: FAMoS: A Flexible and dynamic Algorithm for Model Selection to analyse complex systems dynamics
Source: PLoS Comput Biol. 2019 Aug 16;15(8):e1007230. doi: 10.1371/journal.pcbi.1007230 (PMC6697322; doi:10.1371/journal.pcbi.1007230)
Supplement: S1 Fig — (PDF) [file pcbi.1007230.s001.pdf]

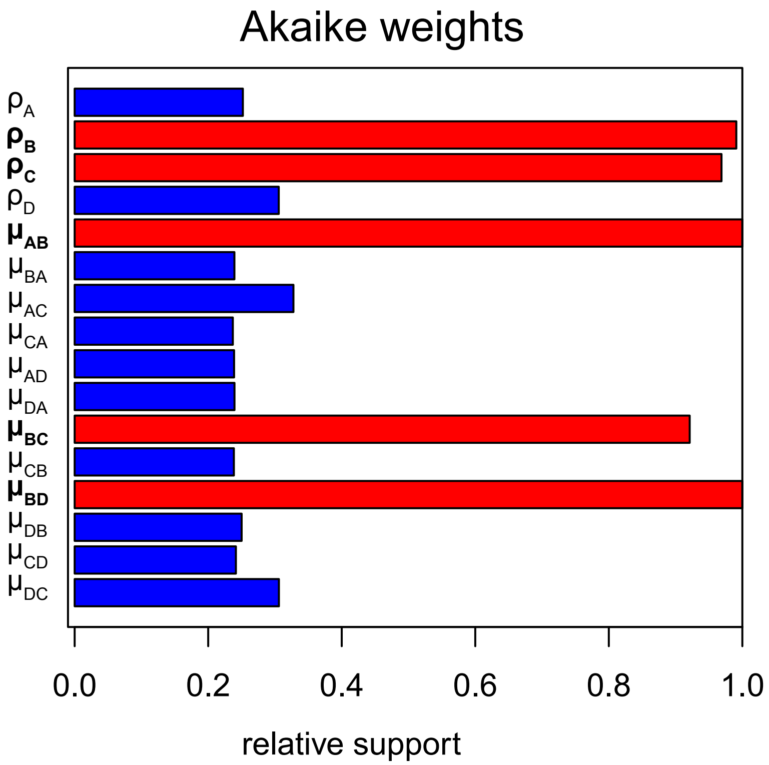

Figure S1: Akaike weights for the individual parameters within the 4-compartment model: Akaike weights over all individual FAMoS runs for the data and models shown in Figure 4B and C. Akaike weights indicate the relative support for the selection of each parameter within the final model based on the performed model evaluations. Parameters belonging to the "true" dynamics, i.e., model 1, are indicated in red and also get the largest support.
