# Supplementary material for: FAMoS: A Flexible and dynamic Algorithm for Model Selection to analyse complex systems dynamics
Source: PLoS Comput Biol. 2019 Aug 16;15(8):e1007230. doi: 10.1371/journal.pcbi.1007230 (PMC6697322; doi:10.1371/journal.pcbi.1007230)
Supplement: S2 Fig — (PDF) [file pcbi.1007230.s002.pdf]

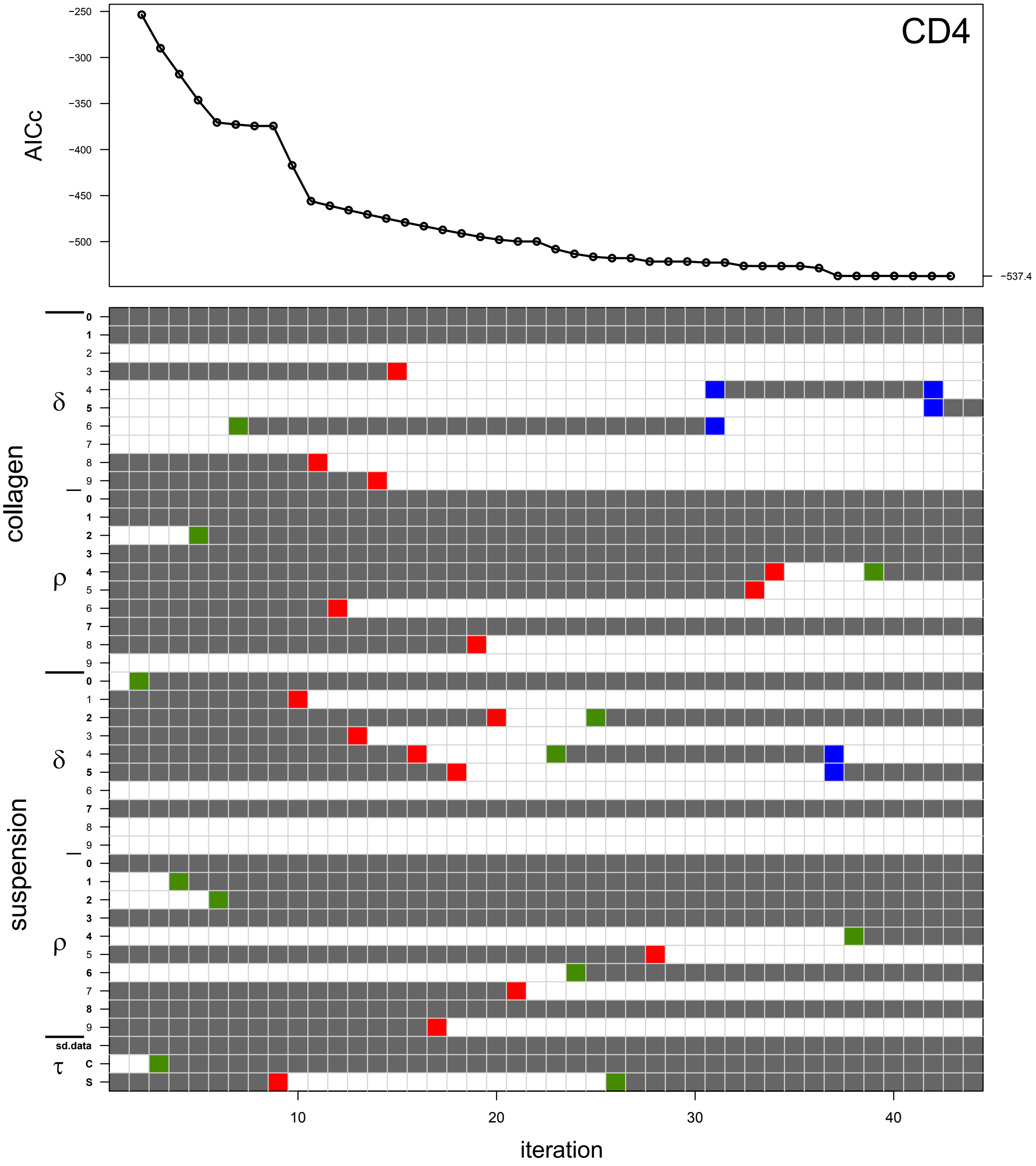

Figure S2: Exemplary FAMoS-run analysing CD4$^+$ T cell dynamics: Visualization of a single FAMoS-run over several iterations with the addition (*forward search*, green), removal (*backward elimination*, red) and swapping (*swap search*, blue) of parameters for analysing the turnover dynamics of CD4$^+$ T cells in suspension and collagen. Parameters that are selected in the final model with the denoted AICc-value of -537.4 are indicated in bold. The final run out of 5 individual FAMoS runs is shown.
