# Supplementary material for: FAMoS: A Flexible and dynamic Algorithm for Model Selection to analyse complex systems dynamics
Source: PLoS Comput Biol. 2019 Aug 16;15(8):e1007230. doi: 10.1371/journal.pcbi.1007230 (PMC6697322; doi:10.1371/journal.pcbi.1007230)
Supplement: S3 Fig — (PDF) [file pcbi.1007230.s003.pdf]

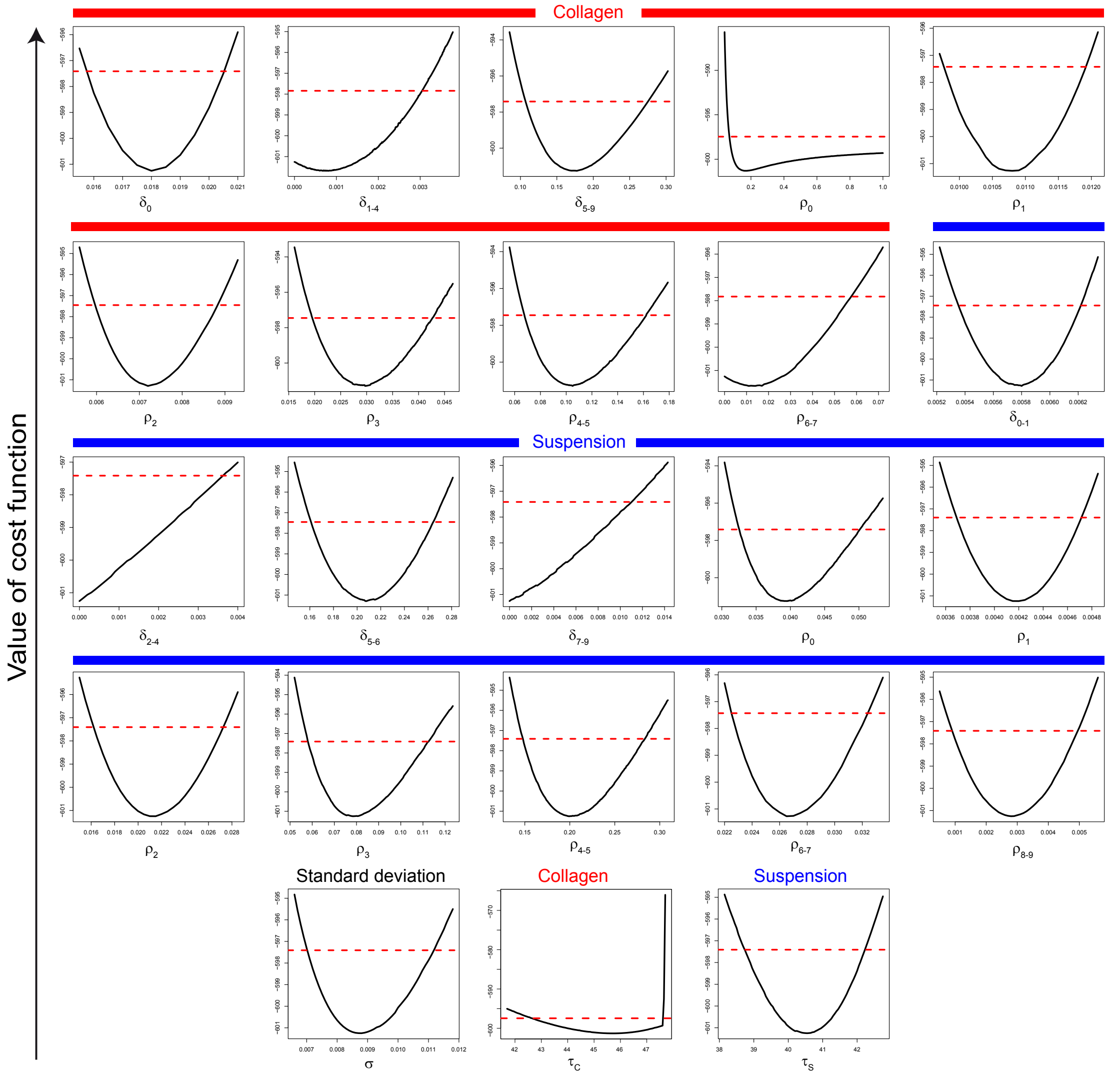

Figure S3: Profile likelihood analysis for parameter estimates of CD4$^+$ T cells: Profile likelihoods for the different proliferation, ρ, and death rates, δ, (each h^{-1}) considered within the model identified for CD4$^+$ T cell turnover dynamics in suspension (blue) and collagen (red). The y-axes show the value of the cost function defined in Eq. (5) (see *Methods*). The last three panels show the profile likelihoods for the estimated standard deviation of the data, σ and the adaptation times for suspension, τ_S, and collagen, τ_C (in hours). Red lines indicate the boundaries of the confidence intervals. Corresponding parameter values are shown in Table 1 in the manuscript.
