# Supplementary material for: FAMoS: A Flexible and dynamic Algorithm for Model Selection to analyse complex systems dynamics
Source: PLoS Comput Biol. 2019 Aug 16;15(8):e1007230. doi: 10.1371/journal.pcbi.1007230 (PMC6697322; doi:10.1371/journal.pcbi.1007230)
Supplement: S5 Fig — (PDF) [file pcbi.1007230.s005.pdf]

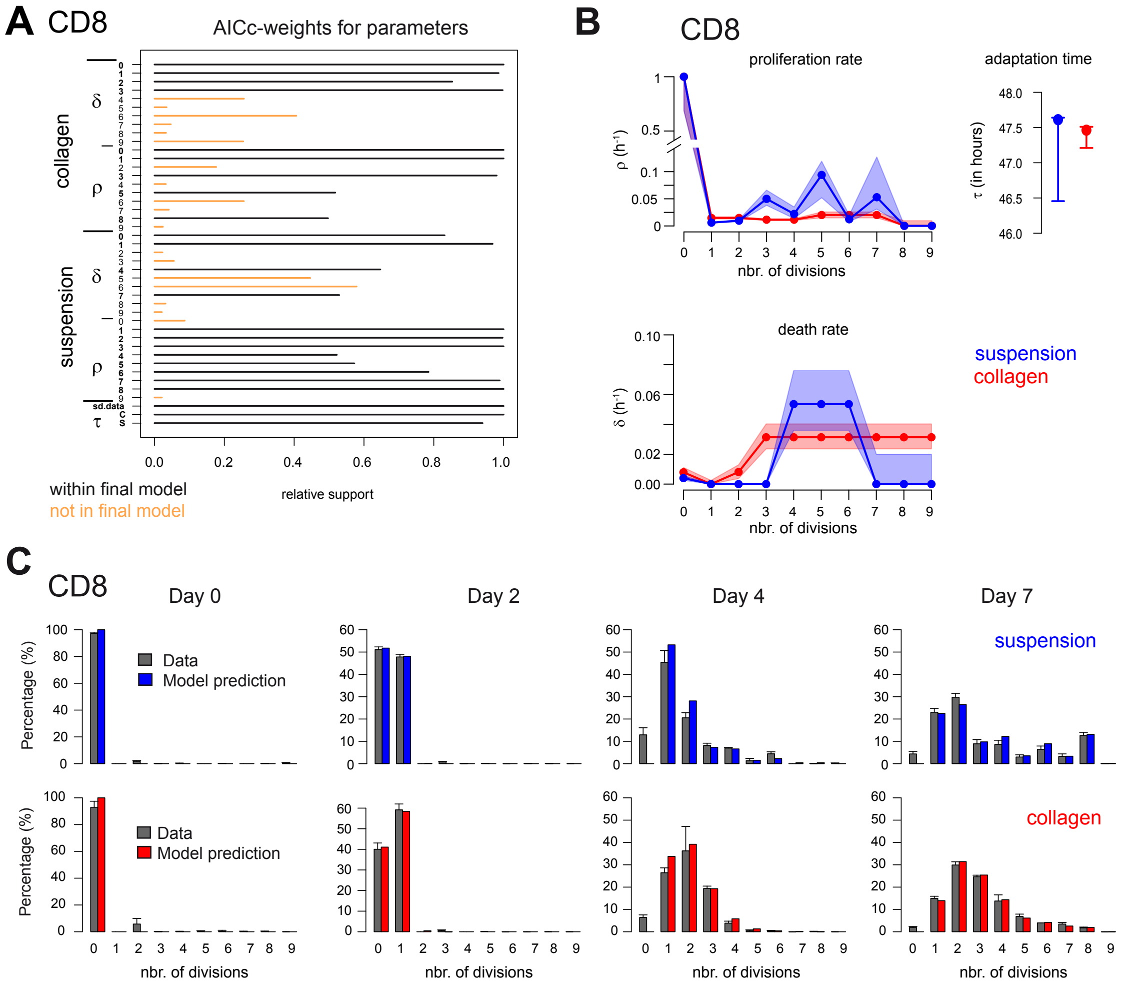

Figure S5: Model selection and parameter estimates for CD8$^+$ T cell dynamics: (**A**) Akaike weights for the individual parameters based on all model evaluations within the 5 independent FAMoS runs performed for the analysis of the CD8$^+$ T cells. Parameters selected within the final model are shown in black, non-selected parameters in orange. (**B**) Parameter estimates for the generation dependent proliferation (ρ) and death (δ) rates, as well as adaptation times (τ) within suspension (blue) and collagen (red). Points define the best estimates with shaded areas indicating the 95% confidence intervals for each parameter determined by profile likelihood analysis (see Table 1 and S6 Figure for individual estimates). (**C**) Observed (grey) and predicted distribution of cell populations for CD8$^+$ T cells for using the best model estimated for each environment.
