# Supplementary material for: FAMoS: A Flexible and dynamic Algorithm for Model Selection to analyse complex systems dynamics
Source: PLoS Comput Biol. 2019 Aug 16;15(8):e1007230. doi: 10.1371/journal.pcbi.1007230 (PMC6697322; doi:10.1371/journal.pcbi.1007230)
Supplement: S7 Fig — (PDF) [file pcbi.1007230.s007.pdf]

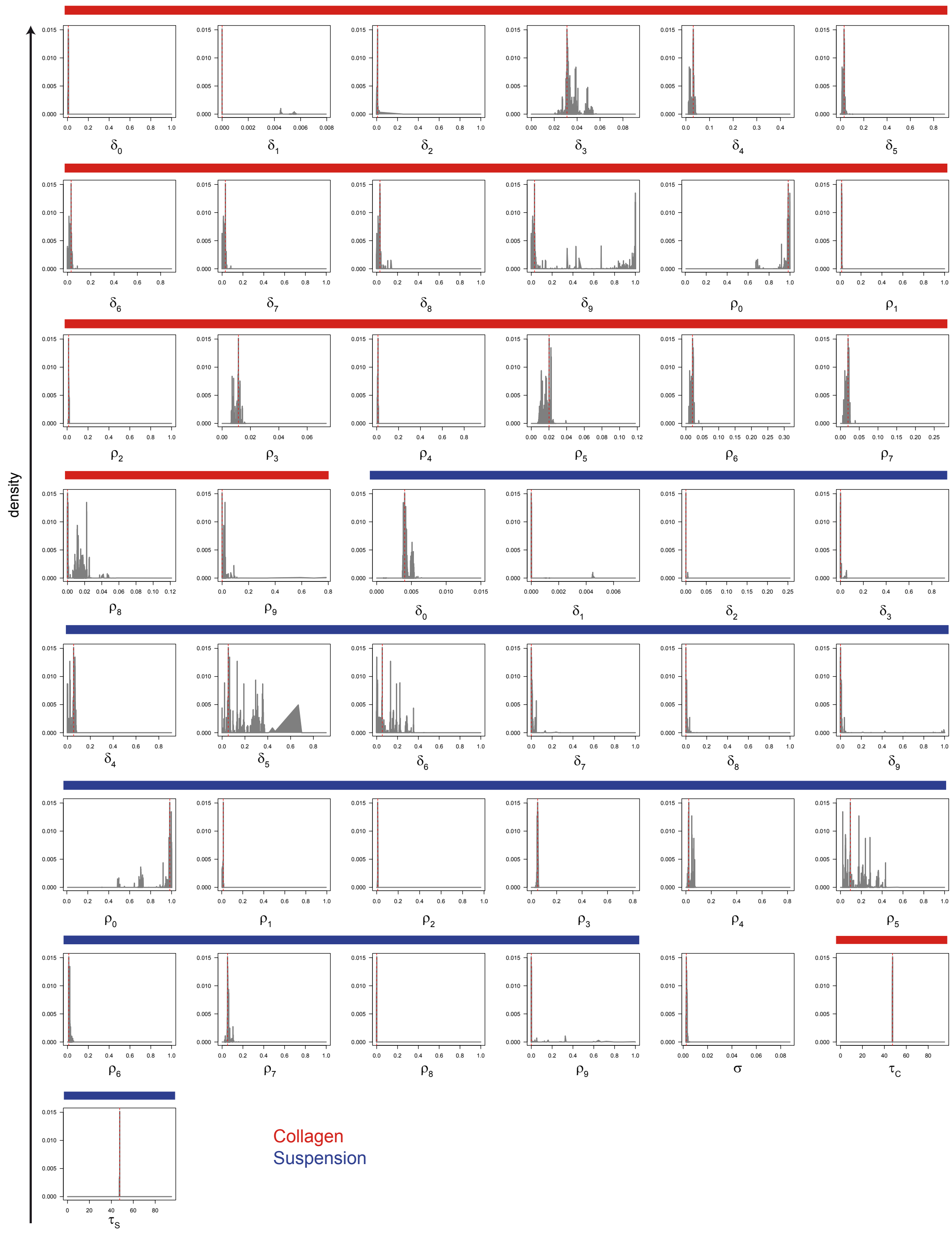

Figure S7: Distribution of parameter estimates for CD8$^+$ T cells considering different model structures: Density plots for individual parameter estimates for the proliferation, ρ, and death rates, δ, (each h^{-1}) considering all models that were evaluated throughout the 5 individual FAMoS-runs with individual parameter estimates weighted by the AIC weights. The red dotted line shows the position of the parameter estimate for the best model which coincides with the parameter having the highest density. Results are shown for suspension (blue) and collagen (red) with the last three panels showing the plots for the estimated standard deviation of the data, σ, and the adaptation times for suspension, τ_S, and collagen, τ_C (in hours).
