# Supplementary material for: FAMoS: A Flexible and dynamic Algorithm for Model Selection to analyse complex systems dynamics
Source: PLoS Comput Biol. 2019 Aug 16;15(8):e1007230. doi: 10.1371/journal.pcbi.1007230 (PMC6697322; doi:10.1371/journal.pcbi.1007230)
Supplement: S8 Fig — (PDF) [file pcbi.1007230.s008.pdf]

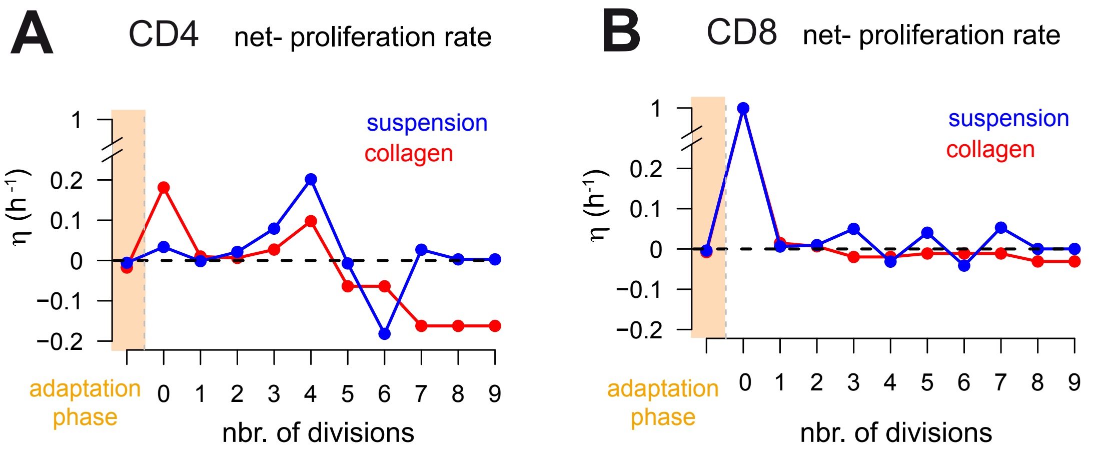

Figure S8: Net-proliferation rates for different generations: Determined net-proliferation rates, $\eta_i = \rho_i - \delta_i$ for each generation $i = 0, 1, \ldots, 9$, for $CD4^+$ (**A**) and $CD8^+$ (**B**) T cells in suspension (blue) and collagen (red). Calculations of the net-proliferation rates are based on the best estimates and shown in S1 Table. During the adaptation phase (orange), the net-proliferation rate is given by the estimated death rate δ_0 in the corresponding culture condition as during the adaptation phase all proliferation rates were set to 0, $\rho_i = 0$, $i = 0, 1, \ldots, 9$.
