# Supplementary material for: FAMoS: A Flexible and dynamic Algorithm for Model Selection to analyse complex systems dynamics
Source: PLoS Comput Biol. 2019 Aug 16;15(8):e1007230. doi: 10.1371/journal.pcbi.1007230 (PMC6697322; doi:10.1371/journal.pcbi.1007230)
Supplement: S1 Appendix — (PDF) [file pcbi.1007230.s009.pdf]

S1 Appendix: FAMoS performance for analysing different model structures - Generalized Linear Models

We evaluated the performance of FAMoS for analysing generalized linear models (GLM) in comparison to the *glmulti*-algorithm within the corresponding `R`-package [1]. To this end, we used the application problem published within [1], which aims at explaining the birth weight of babies given as a binary outcome (low, high) based on 8 predictor variables, i.e., using a binomial GLM. The dataset `birthwt` is part of the *MASS*-package of `R` and the data frame `bwt` is prepared for analysis according to the descriptions made in [1] and [2].

Running the analysis with *glmulti* as specified in [1], a run takes about 1-3 min and returns the best model defined by

```
mdl1 <- low   1 + smoke + ptd + ht + ui + ftv + age + lwt + smoke:ui + age:ftv
```

as given in [1] with an AIC-value of 205.9.

In contrast, running the same analysis with FAMoS (see documentation of FAMoS for the exact code), needs a substantially reduced run time of about 15 s, and returns a slightly different model that provides a better description of the data with an AIC-value of 203.5.

```
mdl2 <- low   1 + age:lwt + smoke:lwt + ptd + ht:age + ui:lwt + ftv + lwt +
                    smoke:ui + age:ftv
```

Re-fitting both models with the standard `glm`-fitting procedure confirms the results, i.e., that the model identified by FAMoS has a better AIC than the model identified by *glmulti*. Thus, the newly established swap-search method improves the model selection process when analysing large model spaces.

1. Calcagno V, de Mazancourt C. glmulti: An R Package for Easy Automated Model Selection with (Generalized) Linear Models. J Statistic Software. 2010;34(12):1–29.
2. Venables WN, Ripley BD. Modern Applied Statistics with S-PLUS. 3rd ed. New York; Berlin; Heidelberg [u.a.]: Springer; 1997.
