# Supplementary material for: FAMoS: A Flexible and dynamic Algorithm for Model Selection to analyse complex systems dynamics
Source: PLoS Comput Biol. 2019 Aug 16;15(8):e1007230. doi: 10.1371/journal.pcbi.1007230 (PMC6697322; doi:10.1371/journal.pcbi.1007230)
Supplement: S1 Table — (PDF) [file pcbi.1007230.s010.pdf]

generation	Suspension		Collagen	
$\eta_i = \rho_i - \delta_i$	**CD4** ($\times 10^{-2}$h^{-1})	**CD8** ($\times 10^{-2}$h^{-1})	**CD4** ($\times 10^{-2}$h^{-1})	**CD8** ($\times 10^{-2}$h^{-1})
η_0	3.36	99.60	18.13	99.21
η_1	-0.16	0.61	0.99	1.47
η_2	2.13	0.97	0.64	0.66
η_3	7.91	4.99	2.71	-1.98
η_4	20.15	-3.15	9.77	-1.98
η_5	-0.73	4.04	-6.39	-1.12
η_6	-18.20	-4.13	-6.39	-1.12
η_7	2.68	5.29	-16.23	-1.12
η_8	0.28	0.01	-16.23	-3.10
η_9	0.28	0.01	-16.23	-3.10

Table S1: Net-proliferation rates for different generations: The calculated net-proliferation rates for each generation with $\eta_i = \rho_i - \delta_i$ and $i = 0, \ldots, 9$ indicating the number of previous divisions. Rates are calculated based on the best estimates obtained for the models identified for each cell type and culture condition (see Table 1 in the main manuscript).
